# Short-term incidence of opioid misuse-related behaviour after elective surgery

**DOI:** 10.1016/j.bja.2026.05.039

**Published:** 2026-07-22

**Authors:** Daniel B. Larach, Miklos D. Kertai, Stephen Bruehl

**Affiliations:** Department of Anesthesiology, Vanderbilt University Medical Center, Nashville, TN, USA

**Keywords:** addiction, opioid misuse, opioids, opioid use disorder, postoperative analgesia

Postsurgical opioid prescribing and associated misuse have received considerable recent attention. Guidelines, state laws, and payor regulations addressing postsurgical opioid prescription amounts are common.^1–3^ NIH funding related to surgical opioid prescribing has exceeded $59 million during the past decade.^4^

Iatrogenic postoperative opioid misuse is important to public health because US surgeons and dentists write 45 million opioid prescriptions yearly,^5^ and >25% of opioid-naïve patients receiving first-time opioid prescriptions obtain them from surgeons or dentists.^6^ Half of patients with more severe pathology such as opioid use disorder (OUD) are introduced to opioids by medical prescriptions written directly to them; many are likely postoperative.^7^

However, rigorous prospective evidence evaluating postoperative opioid misuse incidence is limited. Are postsurgical opioid prescriptions really a problem? Two prospective studies have evaluated opioid misuse-related behaviour in predominantly postoperative cohorts (confusingly, both named ‘STOMP’). One used the validated 17-question Current Opioid Misuse Measure (COMM) to identify an 8.3% misuse-related behaviour incidence in a trauma population 6 months after hospital discharge.^8^ However, 24% of this population did not undergo surgery, and the half-year period between discharge and misuse evaluation makes causation attribution to discharge prescribing challenging. The other study used a two-question non-validated measure to evaluate opioid misuse-related behaviour 1 and 3 months after ambulatory surgery, with a 7.5% overall incidence of misuse-related behaviour in a high-risk (age 15–24 yr) population.^9^ Granular data on specific misuse-related behaviours are not reported in either study.

We combined two prospective postoperative cohorts to conduct a secondary analysis of opioid misuse-related behaviours within the first month after a heterogeneous group of common surgeries.^10,11^ Both cohorts used the nine-item COMM-9 measure of aberrant opioid-related behaviours, which is validated in chronic pain patients taking opioids long-term.^12^ Example items reflect opioid-related behaviours (e.g. taking someone else’s pain medications), cognitions (e.g. thinking about opioids frequently), and negative affect (e.g. thoughts of hurting self). We included all patients in these studies who received a postoperative outpatient opioid prescription and completed the COMM-9 measure within the first postoperative month (Supplementary Fig. S1). The first cohort comprised 187 opioid-naïve adult patients (no opioid prescriptions per electronic medical record query or self-report between 3 months and 14 days preoperatively) from a single-blind, parallel-group, single-centre randomised controlled trial of a ‘nudging’ intervention targeting surgeon opioid prescribing.^11^ A modified COMM-9 querying post-discharge behaviour was assessed once at 14 days by trained research coordinators over the telephone after cholecystectomy, appendicectomy, hysterectomy (non-cancer), urogynaecology surgery, total knee, hip, or shoulder arthroplasty, or 1- or 2-level laminectomy or discectomy without fusion. Opioid consumption was calculated using patient-reported pill counts under real-time telephone guidance from research staff. The second cohort comprised 25 adult elective cardiac surgery patients without daily opioid use during the 30 days before surgery.^10^ COMM-9 was assessed once at 30 days after surgery by trained research coordinators over the telephone, as was opioid consumption through patient-reported pill counts under real-time guidance. Both studies were IRB-approved (#220508, #211530; Vanderbilt University Medical Center). All outcomes were recorded in REDCap.^13,14^ All cases had complete opioid prescribing, consumption, and COMM-9 data.

Our primary objective was to identify opioid misuse-related behaviour incidence (‘moderate’ or ‘high’ COMM-9 risk; probability score ≥28.5) within this combined sample. A secondary objective was to describe specific misuse-related behaviours reported by patients, and an exploratory objective was to examine associations between opioid prescribing, consumption, and misuse-related behaviour incidence. All analyses were conducted using SPSS v.31 (IBM, Armonk, NY, USA).

Patients were primarily older, female, and White (Table 1). More than half underwent total joint arthroplasty (mainly knee and hip), whereas about a quarter had gynaecologic surgery. Nearly all received oxycodone prescriptions after surgery. We identified a combined incidence of opioid misuse-related behaviour of 7.5% (95% confidence interval [CI]: 4.4%–12.0%) within the cohort (16/212 patients; Table 1). Misuse-related behaviour was reported in four of five surgical categories and ranged from 0% (general) to 18.2% (spine), with the caveat that these two categories were the smallest (Supplementary Table S1). Among the 16 patients with ‘positive’ COMM-9 scores, 9 (56%) endorsed taking someone else’s pain medications or using their medication to treat non-pain-related symptoms; nearly all reported spending time thinking about their opioid medications (Supplementary Table S2). Logistic regression assessing the effect on misuse-related behaviour of (1) initial discharge opioid prescription size and (2) percentage of initial discharge opioid prescription consumed (controlling for age, sex, race, and surgery type) found that consuming a greater percentage of the discharge prescription was significantly associated with misuse-related behaviour (adjusted odds ratio [OR] 14.08, 95% CI: 1.90–104.23), as was younger age. Sex, race, surgery type, and discharge prescription size were nonsignificant (Supplementary Table S3).

In a prospective cohort of patients undergoing a heterogeneous group of surgeries, we identified a clinically relevant incidence (7.5%) of behaviours linked to opioid misuse in the context of postoperative outpatient opioid prescribing. We also report specific risk behaviours among these patients. Most patients endorsed COMM-9 items indexing classic misuse behaviour such as taking someone else’s pain medications and treating non-pain-related symptoms with opioids. Consuming a greater percentage of initially prescribed outpatient opioid pills was significantly associated with misuse-related behaviour, providing clinicians with a potential surveillance target requiring additional study in larger cohorts. Such cohorts could more fully assess whether misuse-related behaviour incidences are similar across different surgeries. Earlier literature has identified similar incidence rates, although these previous studies examined heterogeneous surgical/non-surgical cohorts^8^ or used briefer, non-validated misuse measures in a circumscribed age bracket.^9^ We emphasise that opioid misuse-related behaviour is not synonymous with Diagnostic and Statistical Manual of Mental Disorders, Fifth Edition (DSM-5)-defined OUD, but rather represents an early or less-severe OUD-related diagnosis similar to the International Classification of Diseases, 11th Revision (ICD-11) ‘hazardous use of opioids’ diagnosis.^15^

Our study has several limitations. A small absolute number of patients exhibited opioid misuse-related behaviours. COMM-9 has not previously been validated in the postoperative context (including our modification assessing post-discharge behaviour), and its assessment at a single follow-up time point might limit the ability to detect patterns of misuse-related behaviour over time. Because of human subjects protection constraints, we were unable to perform detailed chart reviews to identify baseline risk factors for misuse (e.g. anxiety, depression) or to evaluate the impact of differing intraoperative or postoperative anaesthesia and analgesia protocols. This precluded development of more nuanced risk stratification approaches from our dataset. Our cohort also exhibited limited racial heterogeneity. There were also methodological discrepancies between our two component cohorts, most notably that the first assessed opioid consumption and COMM-9 at 14 days, and the second at 30 days. Finally, our exploratory logistic regression model was limited by the small number of patients developing misuse-related behaviour in the combined cohort, potentially leading to estimation overfitting.

In summary, postoperative opioid misuse-related behaviour occurred in 7.5% of surgical patients assessed using the COMM-9 instrument, a clinically meaningful incidence. To our knowledge, postoperative opioid misuse-related behaviour have not been quantified in a purely surgical adult cohort. A greater percentage consumption of prescribed discharge opioids was associated with increased misuse-related behaviour incidences. These findings suggest that such patients warrant targeted postoperative surveillance, although these exploratory results should be considered hypothesis-generating rather than definitive.

## Supplementary Material

Supplementary Material

## Figures and Tables

**Table 1:** Patient characteristics, opioid data, and opioid misuse-related behaviour risk categorization (COMM-9) across combined cohorts (*n*=212).

Characteristics and opioid data	Mean (SD) or *n* (%)
Age, yr	58.4 (14.3)
Sex, female	127 (59.9%)
Race	
White	178 (84.0%)
African American	22 (10.4%)
Asian	1 (0.5%)
American Indian/Alaska Native	2 (0.9%)
More than one race	3 (1.4%)
Unknown	6 (2.8%)
Surgery category	
General (appendicectomy, cholecystectomy)	13 (6.1%)
GYN (hysterectomy, urogynaecology)	48 (22.6%)
Arthroplasty (total knee, hip, shoulder)	115 (54.2%)
Spine (1–2 level laminectomy/discectomy)	11 (5.2%)
Cardiac	25 (11.8%)
Postoperative discharge date	
Postoperative day 0	144 (67.9%)
Postoperative day ≥1	68 (32.1%)
Postoperative day 1	35 (16.5%)
Postoperative day 2	7 (3.3%)
Postoperative day 3	1 (0.5%)
Unknown*	25 (11.8%)
Postoperative opioid received	
Oxycodone	191 (90.1%)
Hydrocodone	13 (6.5%)
Tramadol	5 (2.4%)
Hydromorphone	2 (0.9%)
Codeine	1 (0.5%)
Initial discharge opioid prescription consumption	
Number of pills	14.7 (13.4)
Percentage of pills	52.6 (35.1)
COMM-9 risk category	*n* (%; 95% CI)
High	7 (3.3; 1.3–6.7)
Moderate	9 (4.2; 2.0–7.9)
No-to-slight	196 (92.5; 88.0–95.6)

* Exact discharge dates were not available for cardiac surgery patients (*n*=25), all of whom were admitted postoperatively. COMM-9 opioid misuse risk categories are based on a validated scoring algorithm; each item (listed in Supplementary Table S2) is weighted differently. For example, endorsing taking someone else’s pain medications is weighted more than seven times greater than endorsing difficulty thinking clearly. 95% confidence intervals (CI) for COMM-9 risk categories were calculated through Clopper-Pearson method.

## Appendix A. Supplementary data

Supplementary data to this article can be found online at https://doi.org/10.1016/j.bja.2026.05.039.
