# Supplementary material for: Short-term incidence of opioid misuse-related behaviour after elective surgery
Source: Br J Anaesth. Author manuscript; Available in PMC 2026 Aug 30. (PMC13525450; doi:10.1016/j.bja.2026.05.039)
Supplement: Supplementary file 1 [file NIHMS2205207-supplement-Supplementary_Material.pdf]

Figure S1: Study flow diagrams

Cardiac surgery study

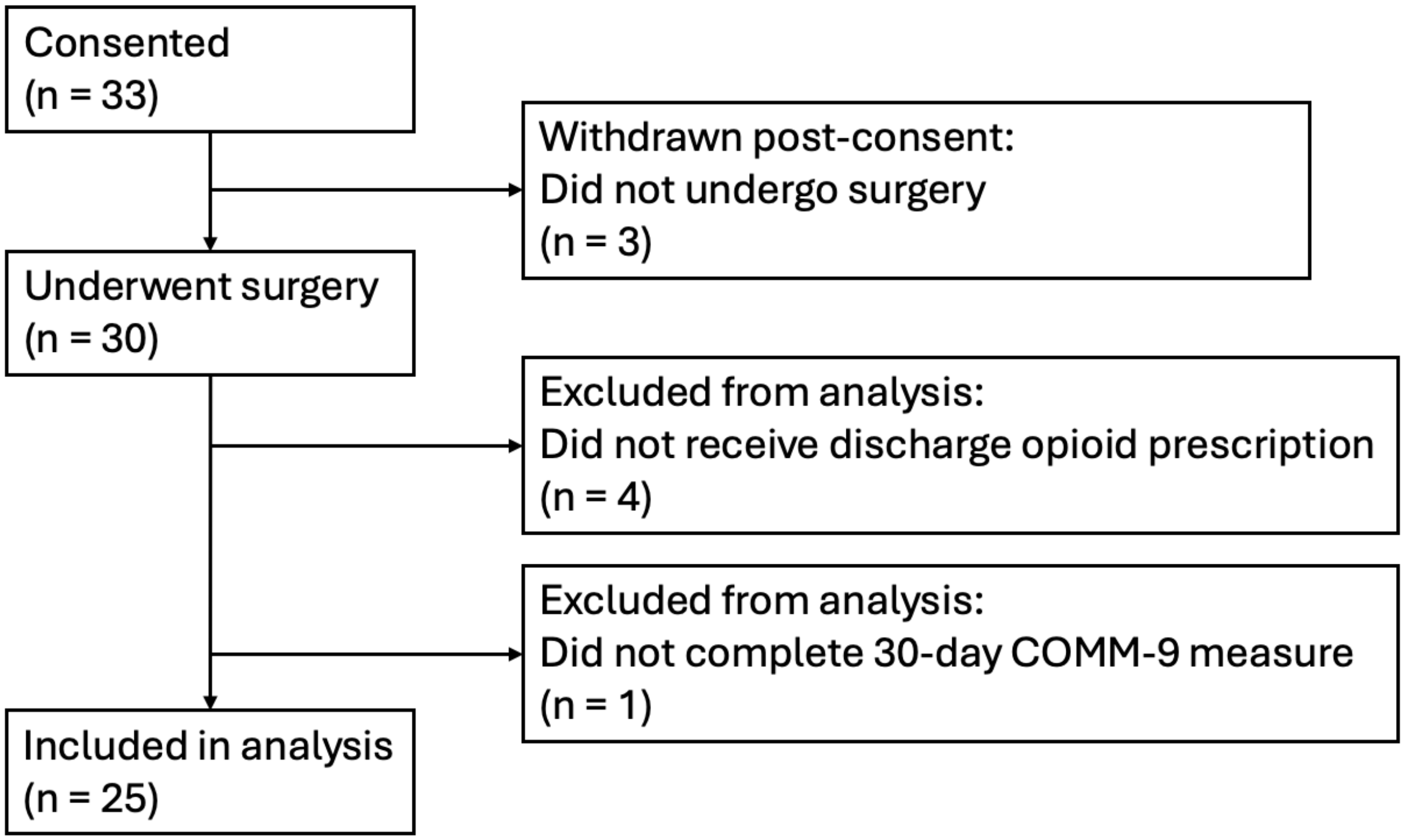

Nudging study

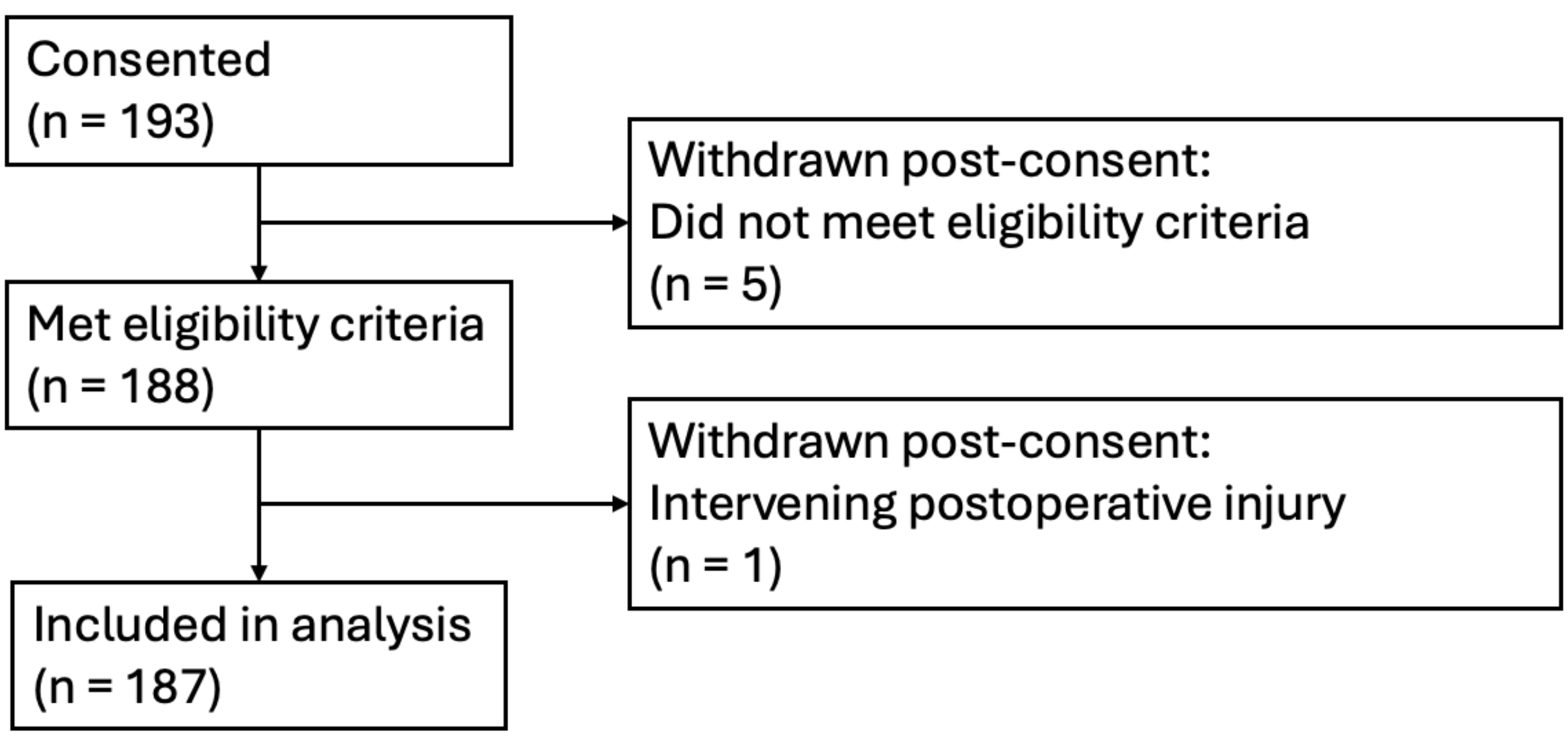

Table S1: COMM-9 opioid misuse-related behavior risk category, stratified by surgery type

	**General**	**Gynecologic**	**Arthroplasty**	**Spine**	**Cardiac**
**Any Misuse-Related Behavior**	0 (0.0%)	2 (4.2%)	9 (7.8%)	2 (18.2%)	3 (12.0%)
High Risk	*0 (0.0%)*	*1 (2.1%)*	*4 (3.5%)*	*1 (9.1%)*	*1 (4.0%)*
Moderate Risk	*0 (0.0%)*	*1 (2.1%)*	*5 (4.3%)*	*1 (9.1%)*	*2 (8.0%)*
**No-to-Slight Misuse-Related Behavior**	13 (100.0%)	46 (95.8%)	106 (92.2%)	9 (81.8%)	22 (88.0%)

Percentages are within each surgery type. “Any Misuse-Related Behavior” encompasses patients reporting moderate or greater misuse-related behavior risk. Surgeons were responsible for all opioid prescribing to patients.

Table S2: Specific opioid misuse-related behaviors endorsed by individuals in elevated COMM-9 risk categories (n=16)

**COMM-9 item**	**Number (%)**
1. Trouble thinking clearly/memory problems	12 (75.0%)
2. Went to other physicians for pain medication	2 (12.5%)
3. Thoughts of hurting self	1 (6.3%)
4. Time spent thinking about opioid medications (having enough, taking them, dosing schedule)	15 (93.8%)
5. Took someone else's pain medications	5 (31.3%)
6. Getting angry with people	9 (56.3%)
7. Took more medication than prescribed	1 (6.3%)
8. Used pain medication for non-pain symptoms (sleep, mood, stress)	7 (43.8%)
9. Visited emergency room	1 (6.3%)

Each COMM-9 item is scored 0-4 ("never", "seldom", "sometimes", "often", "very often"). We report non-zero item scores from patients scoring in the "high" and "moderate" COMM-9 risk categories.

Table S3: Exploratory logistic regression assessing relationship between incidence of COMM-9-indexed misuse-related behavior, percentage of postoperative outpatient opioid pills consumed, and number of postoperative outpatient opioid pills prescribed (controlling for age, sex, race, and surgery type)

	**B**	**S.E.**	***p*-value**	**Adjusted odds ratio**	**95% C.I.**
Predictors					
Initial Discharge Opioid Prescription Size	0.008	0.029	0.786	1.008	0.952-1.068
Percentage of Initial Discharge Opioid Prescription Consumed	2.645	1.021	**0.010**	**14.078**	**1.901-104.229**
Covariates					
Age	-0.059	0.024	**0.014**	**0.943**	**0.900-0.988**
Sex (Male Ref.)	-1.502	0.790	0.057	0.223	0.047-1.048
Race:					
White (Ref.)			0.990		
American Indian/Alaska Native	-17.880	27271	NC		
Asian	-18.509	40192	NC		
African American	-0.867	1.154	0.453	0.420	0.044-4.039
>1 Race	-19.449	22161	NC		
Unknown/Not Reported	-18.635	14821	NC		
Surgery Category:					
Arthroplasty (Ref.)			0.496		
General	-18.595	10101	NC		
GYN	-0.886	1.418	0.532	0.412	0.026-6.633
Spine	1.557	1.049	0.138	4.744	0.607-37.108
Cardiac	0.014	0.853	0.987	1.014	0.191-5.394

The dependent variable was report of moderate or greater misuse-related behavior risk, indexed by COMM-9. Logistic regression was used due to failure of parallel-lines test during ordinal regression, most likely due to small number of patients in the “moderate” and “high” risk categories. Significance defined as two-tailed *p*-value <0.05, bolded in table. *P*-values, adjusted odds ratios and 95% confidence intervals for several race and surgery categories were non-calculable (denoted “NC”) due to sparse data (small subgroup sizes).

“Percentage of Initial Discharge Opioid Prescription Consumed” was coded as a proportion (0-1); patients consuming 100% of their discharge prescription had 14.078 times the odds of opioid misuse-related behavior compared to those consuming 0% of their prescription (with the caveat of a very broad 95% CI). Adjusted odds ratios for other variables were coded as follows: “Initial Discharge Opioid Prescription Size”: per one additional opioid pill prescribed; Age: per one year increase in age. Reference categories for sex, race, and surgery category are listed in the table, above.
